# Acute Supraventricular Tachycardia Revealing a Massive Saddle Pulmonary Embolism in a Young Male With Rhinovirus Infection: A Case Report

**DOI:** 10.7759/cureus.95168

**Published:** 2025-10-22

**Authors:** Mohammed Y Ishag, Hassan A Mohammed, Mohammed A Alfadl, Mohammed S Ali, Hafsa Y Mahammed

**Affiliations:** 1 Internal Medicine, Sudan Medical Specialization Board (SMSB), Khartoum, SDN; 2 Internal Medicine, Nizwa Hospital, Nizwa, OMN; 3 General Practice, Sudan Medical Council, Khartoum, SDN

**Keywords:** catheter-directed thrombolysis (cdt), computed tomography pulmonary angiography, pulmonary embolism (pe), supraventricular tachycardia (svt), venous thromboembolism (vte)

## Abstract

Pulmonary embolism (PE) is a life-threatening cardiovascular emergency that can present with a broad spectrum of clinical manifestations. While sinus tachycardia is the most common rhythm disturbance associated with acute PE, supraventricular tachycardia (SVT) as the initial presentation is distinctly uncommon, especially in otherwise healthy individuals without predisposing risk factors. We report the case of a 37-year-old previously healthy male who presented to the emergency department with acute palpitations, chest tightness, and shortness of breath following a short viral prodrome. On arrival, he was in hemodynamically unstable SVT that was resistant to pharmacological therapy and required synchronized direct current cardioversion. Initial echocardiography demonstrated right ventricular dilatation and dysfunction, and subsequent computed tomography pulmonary angiography revealed a massive saddle pulmonary embolism with bilateral extension. The patient was admitted to the high dependency unit and started on therapeutic anticoagulation and supportive therapy. During admission, he developed recurrent and refractory episodes of SVT, ultimately necessitating systemic thrombolysis with intravenous alteplase. Arrhythmia control was achieved with digoxin and bisoprolol, and follow-up echocardiography showed normalization of right ventricular function. He was discharged after seven days in stable condition on lifelong oral anticoagulation and beta-blocker therapy, with outpatient follow-up arranged. This case highlights SVT as a rare but clinically significant presentation of acute PE, underscoring the importance of considering PE in patients with new-onset tachyarrhythmias and hemodynamic compromise. Early recognition and timely management are critical to preventing morbidity and mortality in such presentations.

## Introduction

Pulmonary embolism (PE) is a common and potentially fatal condition, with presentations ranging from mild dyspnoea to circulatory collapse. Classic symptoms include chest pain, shortness of breath, and syncope, yet the clinical spectrum is highly variable. Arrhythmias may complicate acute PE, most often due to right ventricular strain, but supraventricular tachycardia (SVT) as the primary presenting feature is distinctly uncommon and may obscure the underlying diagnosis [1,2]. Discrete supraventricular tachycardia (SVT) is much less frequently described in the literature.

The recognition of atypical presentations is especially critical in young patients without traditional risk factors or comorbidities, where the index of suspicion for PE is usually lower [3]. In such individuals, delayed diagnosis can increase morbidity and mortality, particularly in massive or saddle embolism.

The pathophysiologic basis for arrhythmias in PE is multifactorial, including ventricular strain, hypoxia, and catecholamine surge. Infections, meanwhile, are well recognized as transient prothrombotic triggers through systemic inflammation and endothelial activation, yet their contribution outside of severe viral illnesses such as influenza or COVID-19 remains underappreciated [4].

This case combines both unusual phenomena-SVT as an initial manifestation of massive PE and an acute viral infection as a potential thrombotic trigger-highlighting the need for diagnostic vigilance. This report aims to raise awareness of SVT as an initial sign of PE in otherwise healthy individuals, underscoring the importance of considering PE even in atypical cardiac presentations.

## Case presentation

A 37-year-old Pakistani male garage worker, with no significant past medical or family history, presented to the emergency department with an acute onset of palpitations, chest tightness, and shortness of breath. He had a severe sore throat, intermittent dry cough, recurrent vomiting, and epigastric discomfort for the last four days. No history of fever, haemoptysis, jaundice, leg swelling, recent travel, or contact with sick individuals, and he was not on any regular medications.

On arrival, he was conscious but restless. Vital signs showed heart rate (HR) 190 bpm (regular), blood pressure (BP) 90/45 mmHg, respiratory rate (RR) 25/min, peripheral oxygen saturation (SpO₂) 80% on room air, and afebrile. Examination revealed clear lungs, normal heart sounds without murmurs, and mild epigastric tenderness.

**Figure 1:**
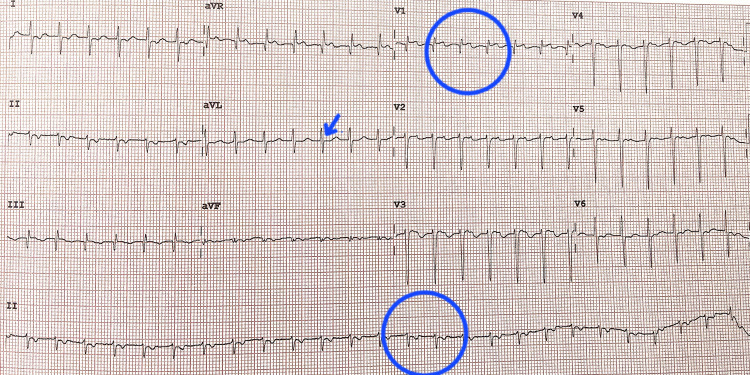
ECG showing supraventricular tachycardia with narrow QRS complexes. Electrocardiogram (ECG) recorded at a paper speed of 25 mm/s and amplitude of 10 mm/mV, showing regular R–R intervals with a ventricular rate of 155 beats per minute, narrow QRS complexes, and absence of visible P waves (blue arrow), consistent with supraventricular tachycardia (SVT).

**Figure 2:**
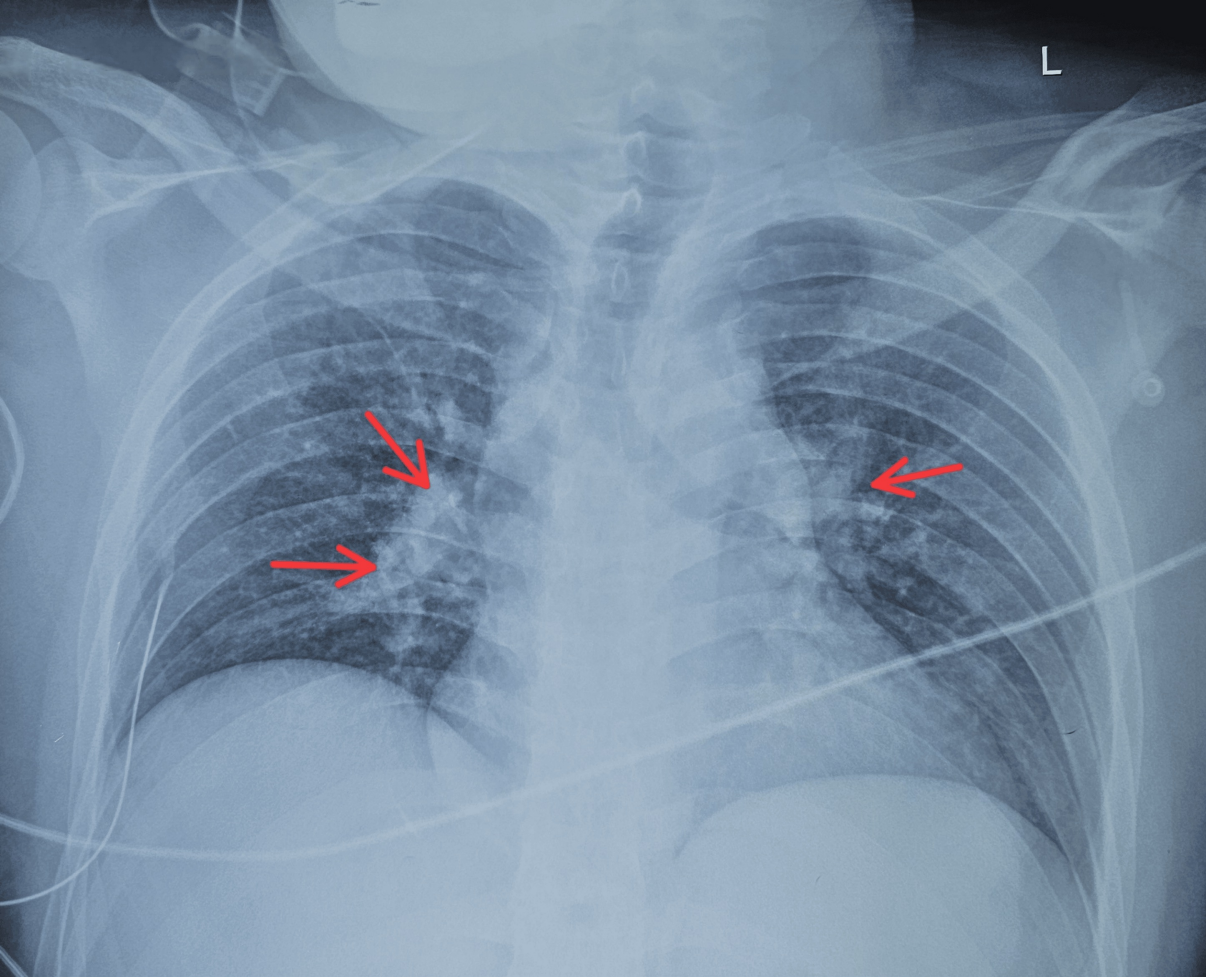
Chest X-ray showing bilateral perihilar haziness (red arrows)

Electrocardiogram (ECG) showed regular narrow-complex SVT at 190 bpm, with absent visible P waves and a short RP interval, consistent with typical AV nodal reentrant tachycardia (AVNRT) (Figure 1). Initial attempts at vagal maneuvers, intravenous adenosine, and verapamil failed. Synchronized direct current cardioversion (50 J) restored sinus rhythm at 116 bpm, with normal P wave morphology and PR interval. Chest radiography showed perihilar haziness more on the right side (Figure 2).

**Figure 3:**
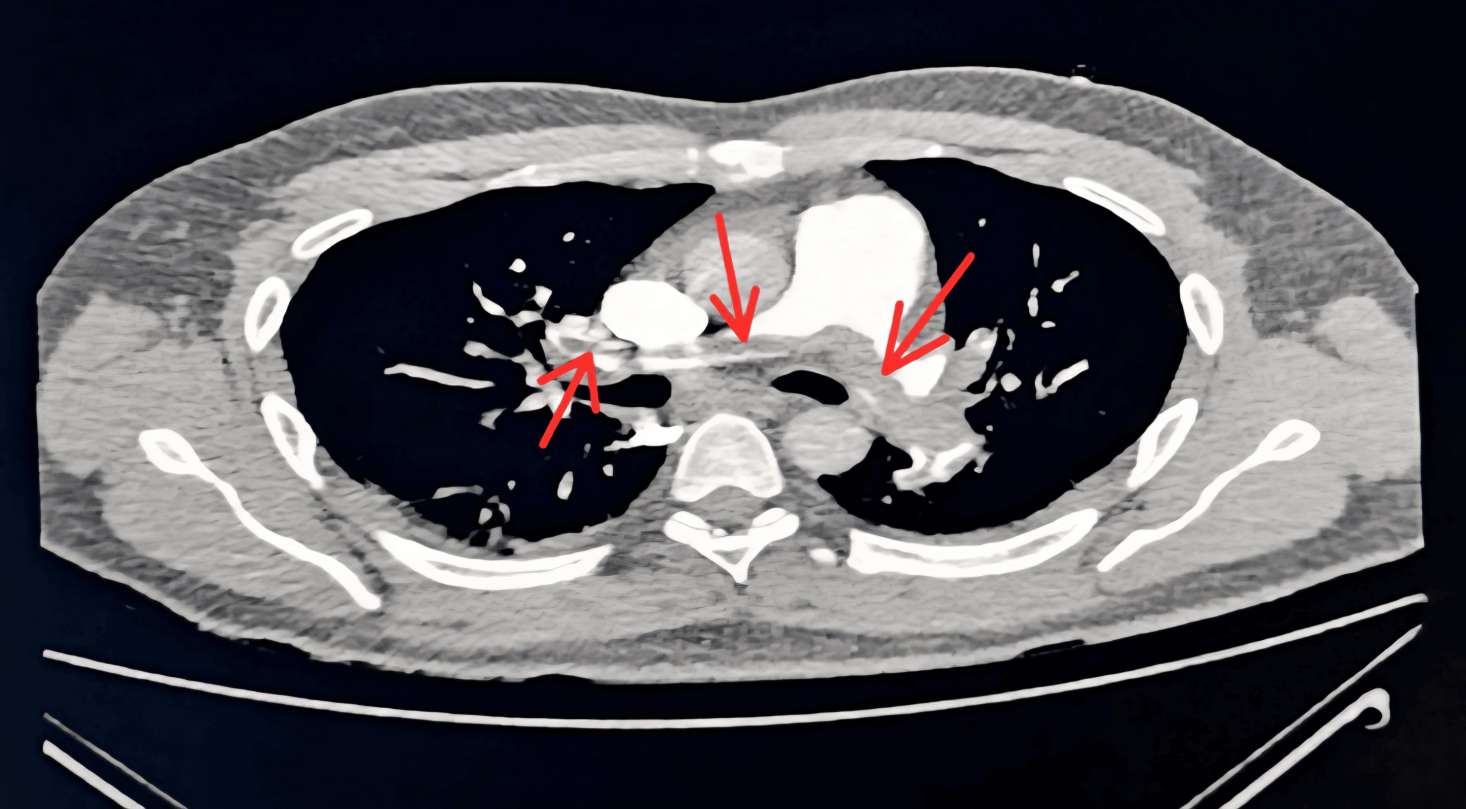
Chest CT scan showing a massive saddle pulmonary embolism. CT Pulmonary Angiogram showing a large saddle-shaped thrombus involving the right and left main pulmonary arteries with extension into segmental and subsegmental branches, consistent with massive pulmonary embolism

Bedside echocardiography revealed a dilated right atrium and ventricle, global left ventricular hypokinesia (ejection fraction (EF) 40-45%), paradoxical septal motion, grade I diastolic dysfunction, mild-to-moderate tricuspid regurgitation, and moderate pulmonary arterial hypertension. CT Pulmonary Angiography confirmed a large saddle thrombus involving the main pulmonary arteries with extension into segmental branches, consistent with massive pulmonary embolism (Figure 3).

He was diagnosed with intermediate- to high-risk, unprovoked PE in the context of an acute upper respiratory tract infection. Initial management included therapeutic low-molecular-weight heparin (LMWH), high-flow oxygen, cautious IV fluids, intravenous ceftriaxone, and analgesia. Catheter-directed thrombolysis (CDT) and thrombophilia screening were considered but declined due to financial constraints. Initial laboratory investigations are summarized in Table 1.

**Table 1:** Initial laboratory investigations ^*^Bold values indicate abnormal results. Hb: hemoglobin; TWBCs: total white blood cells; PLT: platelet; CRP: C-reactive protein; ALT: alanine aminotransferase; AST: aspartate aminotransferase; INR: international normalised ratio; Pro-BNP: N-terminal pro–B-type natriuretic peptide; HAV: hepatitis A virus; HBV: hepatitis B virus; HCV: hepatitis C virus; HEV: hepatitis E virus; HIV: human immunodeficiency virus; RVP: respiratory viral panel; Rhinovirus RNA: rhinovirus ribonucleic acid; COVID-19 Ag test: coronavirus disease 2019 antigen test; PaO2: partial pressure of oxygen; PaCO2: partial pressure of carbon dioxide.

Investigations	Results^*^	Normal values
Hb	14.4	14-16.5 g/dL
TWBCs	26.7	4-11 x 10⁹/L
Neutrophils	22.1	2.5-8 x 10⁹
Lymphocytes	2.63	1.2-4 x 10⁹
PLT	135	150-400 x 10⁹/L
CRP	105.2	0-5 mg/l
Urea	9.5	2.5-7.8 mmol/L
Creatinine	133.3	44-97 µmol/L
Bilirubin	39.2	5.1-17 µmol/L
AST	2454	10-40 IU/L
ALT	1664	10-55 IU/L
INR	1.67	0.8-1.1
Troponin	391.9	3.7-34.2 pg/ml
Pro BNP	5017	11.3-124.9 pg/ml
Serum amylase	73.00	25-125 u/l
Nasopharyngeal RVP	Rhinovirus RNA +	-ve
COVID-19 Ag test	-ve	-ve
PH	7.34	7.34-7.45
PaO2	35.9	75-89 mmHg
PaCO2	40.7	35-45 mmHg
Lactate	4.4	0-2 mmol/L
Blood and urine culture	No growth	No growth	
HAV/HBV/HCV/HEV/HIV	-ve	-ve

The patient developed recurrent SVT at 180-200 bpm. Systemic thrombolysis with intravenous alteplase was administered within 48 hours of admission to our hospital. The tachyarrhythmia remained resistant to adenosine and amiodarone and was temporally associated with right heart strain from pulmonary embolism. Arrhythmia was ultimately controlled with digoxin and bisoprolol (7.5 mg daily) under close monitoring, without any observed adverse effects.

By day 5, the patient was asymptomatic, successfully weaned off supplemental oxygen, and free of tachyarrhythmias. Laboratory parameters normalized, and repeat echocardiography demonstrated resolution of right ventricular dilation with improved cardiac function. Differential diagnoses considered included viral myocarditis and thyroid disease, which were excluded based on investigations and clinical course.

After a total of 7 days in hospital, he was discharged on lifelong oral anticoagulation with rivaroxaban and bisoprolol, with counseling on medication adherence, travel precautions, and scheduled follow-up in general medicine and cardiology clinics.

## Discussion

This case illustrates an uncommon clinical scenario: acute supraventricular tachycardia (SVT) as the presenting manifestation of a massive saddle pulmonary embolism (PE) in a young, previously healthy adult. While sinus tachycardia is the most frequent rhythm disturbance in PE, discrete atrial tachyarrhythmias such as SVT are unusual and may delay diagnosis when attributed to primary cardiac causes. Case reports and small series have documented SVT or paroxysmal supraventricular tachycardia as initial clues to PE, underscoring that arrhythmia may occasionally be the only or dominant manifestation [5,6].

The pathophysiology involves acute elevation of pulmonary vascular resistance and right ventricular (RV) afterload, leading to RV dilatation, wall stress, ischemia, and autonomic activation, which can trigger atrial irritability and re-entrant arrhythmias. ECG patterns of RV strain - including atrial arrhythmias - are associated with early clinical deterioration in PE and should prompt urgent risk stratification [7]. Sustained or recurrent SVT in the context of dyspnoea and hypoxemia should therefore raise suspicion for PE, particularly when supported by echocardiographic evidence of RV dysfunction.

Epidemiologic data suggest that respiratory infections may act as transient prothrombotic triggers. Studies show an increased risk of venous thromboembolism (VTE) following hospitalization for acute respiratory illness, including both influenza and non-COVID viral infections [8]. Mechanistic work further demonstrates that respiratory viruses can promote systemic and local procoagulant activity, such as induction of tissue factor-bearing microparticles [9]. Although direct clinical evidence linking rhinovirus to acute PE is sparse, it is biologically plausible that viral infection contributed to thrombogenesis in this patient, though causality remains speculative.

From a management perspective, this patient had intermediate- to high-risk PE with hemodynamic compromise and RV dysfunction, complicated by recurrent SVT. Current guidelines recommend systemic thrombolysis for high-risk (hemodynamically unstable) PE, while in intermediate- to high-risk patients with clinical deterioration or refractory RV dysfunction, escalation to reperfusion therapy (systemic thrombolysis or catheter-directed therapies) is appropriate [7,10,11]. In this case, systemic thrombolysis was life-saving given the patient’s instability and resource constraints. Catheter-directed therapy and thrombophilia screening were considered but declined for financial reasons, reflecting real-world limitations in PE management.

Control of arrhythmia required multiple strategies: vagal maneuvers, adenosine, verapamil, and electrical cardioversion provided only temporary benefit, while durable rhythm control was achieved after systemic thrombolysis reduced RV strain. Digoxin and bisoprolol stabilized the patient thereafter. This emphasizes that resolution of right heart strain through reperfusion is often necessary for definitive arrhythmia control in PE [12,13]. In addition, we considered differential mechanisms of arrhythmia: while our ECG features (short RP, absent P waves) favored atrioventricular nodal reentrant tachycardia (AVNRT), atrial tachycardia remains a potential alternative in some cases, and awareness of both mechanisms may aid initial recognition in emergency settings.

A previously published case by Timol et al. described a patient with SVT secondary to PE in the context of a superior vena cava thrombus, illustrating a similar presentation of atrial tachyarrhythmia as a clue to occult pulmonary embolism [14]. This reinforces that atrial arrhythmias, though uncommon, should prompt consideration of PE, especially in atypical presentations.

In summary, this case illustrates that supraventricular tachycardia (SVT) can present as an initial manifestation of acute pulmonary embolism (PE), even in young patients without traditional comorbidities, emphasizing the importance of maintaining clinical vigilance for PE in cases of unexplained or sustained tachyarrhythmias [5,6]. Additionally, respiratory infections may serve as transient prothrombotic triggers contributing to VTE; while mechanistic evidence supports this association, direct evidence involving rhinovirus infection remains limited. Finally, in intermediate- to high-risk PE cases complicated by hemodynamic deterioration or refractory arrhythmias, timely reperfusion therapy - whether systemic thrombolysis or catheter-directed intervention - can be life-saving, and decisions should be individualized according to patient risk profile, available resources, and clinical context [7,10,11].

## Conclusions

This case highlights the rare but clinically significant coexistence of supraventricular tachycardia (SVT) and acute pulmonary embolism (PE) in an otherwise healthy young adult. The presence of persistent tachyarrhythmia in the setting of right ventricular strain can complicate hemodynamic stability, mask underlying pathology, and delay definitive diagnosis. Prompt recognition, systematic evaluation, and early initiation of appropriate anticoagulation and reperfusion therapy were essential for a favourable outcome.

Clinicians should maintain a high index of suspicion for pulmonary embolism in patients presenting with new-onset, resistant SVT - particularly when accompanied by right ventricular strain or hypoxemia - even in the absence of traditional risk factors. In hemodynamically unstable cases, early imaging for PE should be prioritized to avoid missed or delayed diagnosis.
